# Rapid assays of SARS-CoV-2 virus and noble biosensors by nanomaterials

**DOI:** 10.1186/s40580-023-00408-z

**Published:** 2024-01-08

**Authors:** Yang Liu, Yilong Li, Yuteng Hang, Lei Wang, Jinghan Wang, Ning Bao, Youngeun Kim, Ho Won Jang

**Affiliations:** 1 School of Public Health, Nantong University, Nantong, 226019 Jiangsu People’s Republic of China; 2 Department of Materials Science and Engineering, Research Institute of Advanced Materials, Seoul National University, Seoul, 08826 Republic of Korea; 3 NantongEgens Biotechnology Co., LTD, Nantong, 226019 Jiangsu People’s Republic of China

**Keywords:** SARS-CoV-2 virus, Rapid assays, Biosensors, Nanomaterials

## Abstract

The COVID-19 outbreak caused by SARS-CoV-2 in late 2019 has spread rapidly across the world to form a global epidemic of respiratory infectious diseases. Increased investigations on diagnostic tools are currently implemented to assist rapid identification of the virus because mass and rapid diagnosis might be the best way to prevent the outbreak of the virus. This critical review discusses the detection principles, fabrication techniques, and applications on the rapid detection of SARS-CoV-2 with three categories: rapid nuclear acid augmentation test, rapid immunoassay test and biosensors. Special efforts were put on enhancement of nanomaterials on biosensors for rapid, sensitive, and low-cost diagnostics of SARS-CoV-2 virus. Future developments are suggested regarding potential candidates in hospitals, clinics and laboratories for control and prevention of large-scale epidemic.

**Graphical Abstract:**

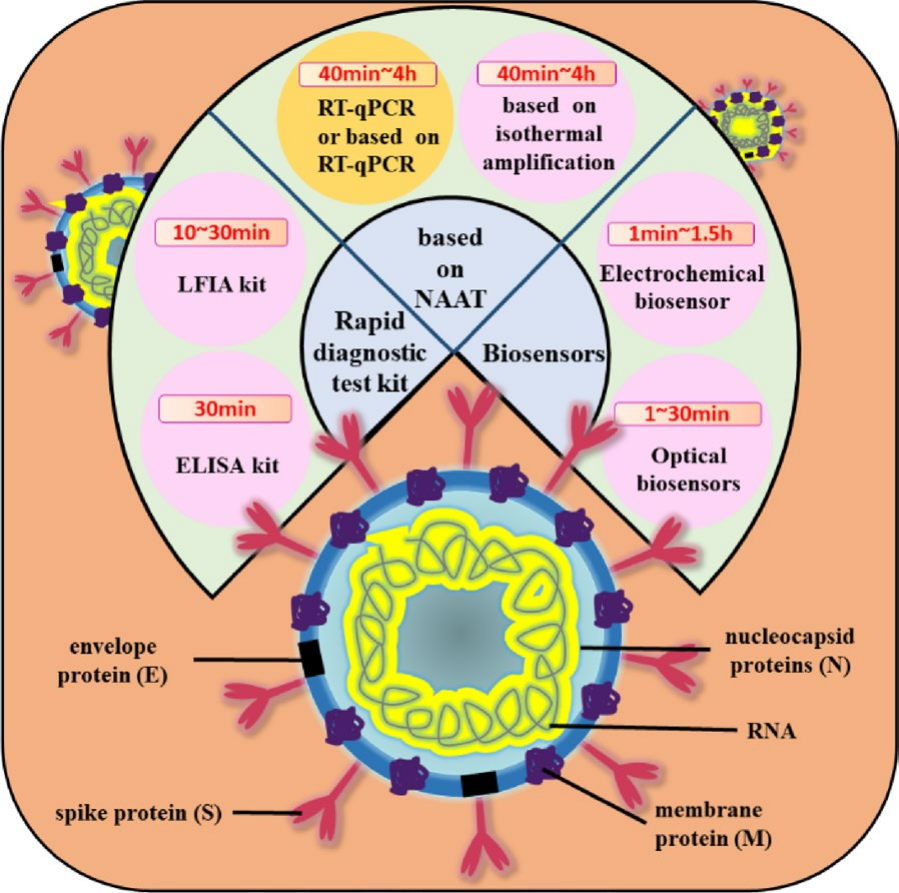

## Introduction

COVID-19 is an infectious disease caused by the SARS-CoV-2 coronavirus. The global excess mortality associated with COVID-19 was estimated to be 14.91 million, suggesting 9.49 million more deaths than those globally reported as directly attributable to COVID-19 [1]. Public health and social measures have been implemented across the world to reduce SARS-CoV-2 transmission, morbidity, and mortality from COVID-19 and to prevent the overburdening of the health systems and other critical social functions. SARS-CoV-2 primarily affects the respiratory system [2] with associated symptoms such as fever, cough, expectoration, headache, myalgia, or fatigue. Individuals with asymptomatic and atypical clinical manifestations contribute factors to complicate disease transmission [3]. SARS-CoV-2 also may cause severe pneumonia and acute respiratory distress syndrome [4]. It is worth noting that in addition to the respiratory system, SARS-CoV-2 damages the cardiovascular system, the endocrine system, and the reproductive system [5]. Previous investigations have suggested that manifestations of cardiovascular disease are a significant cause of mortality [6]. In the reproductive system, extensive studies have shown that SARS-CoV-2 can affect male serum testosterone, fertility, sexual function [7–9] and female ovarian function as well as pregnancy [10–12]. Recent evidence supported that SARS-CoV-2 could also affect the urinary tract [13], and neuropsychiatric symptoms [14]. Other reports also implied association of COVID-19 with digestive disorders [15] and Alzheimer’s disease [16]. Moreover, patients with COVID-19 may also experience eye symptoms such as dry eyes, conjunctival hyperemia, and conjunctival congestion [17]. At the same time, the COVID-19 stigmatization also brought various long-term complications and sequelae [18], even additional pain to patients [19]. It was also observed that psychological symptoms including anxiety, depression, and post-traumatic stress disorder have an association with post-COVID-19 [20, 21]. Despite worldwide efforts to contain the spread of SARS-CoV-2, the COVID-19 pandemic continued as the virus evolved into several variants and mutants [22]. When it comes to SARS-CoV-2 detection, SARS-CoV-2 in wastewater poses a high health risk to human beings [23], and wastewater surveillance becomes a vital part of the assessment and detection of SARS-CoV-2 [24, 25]. Hence, it could be of great significance to detect SARS-CoV-2 for assessment of risks and epidemiology of infectious diseases as well as the development of new responses to combat pathogens in the future [26].

To date, there are two general types of rapid tests available for COVID-19, namely, serological tests and nucleic acid-based tests. While serological detection has the advantages of being easier to conduct without need for sophisticated instruments, they highly depend on antibody detection, which requires seroconversion to occur in patients prior to administration of the test. Amongst nucleic acid-based tests, reverse transcription-quantitative polymerase chain reaction (RT-qPCR) is still the golden standard for the detection of SARS-CoV-2 with limitations such as being time-consuming and causing false negatives. Recent evidence suggested that individuals tested with typical symptoms but showed negative in RT-qPCR results had a high likelihood of actually being infected with COVID-19 [27–29]. Countless factors influence the detection of SARS-CoV-2 using RT-qPCR, such as disease staging, sample collection methodology and sample storage, RNA extraction methodologies, choice of different SARS-CoV-2 targets, maximum Cycle Threshold (Ct), primer–probe dimerization occurrence, etc. [30] Furthermore, having a point mutation in the SARS-CoV-2 N gene (e.g., G29195T) may result in false-negative SARS-CoV-2 RT-qPCR results [31]. Therefore, diagnostic tools that could rapidly detect COVID-19 play critical roles in combating SARS-CoV-2.

**Fig. 1:**
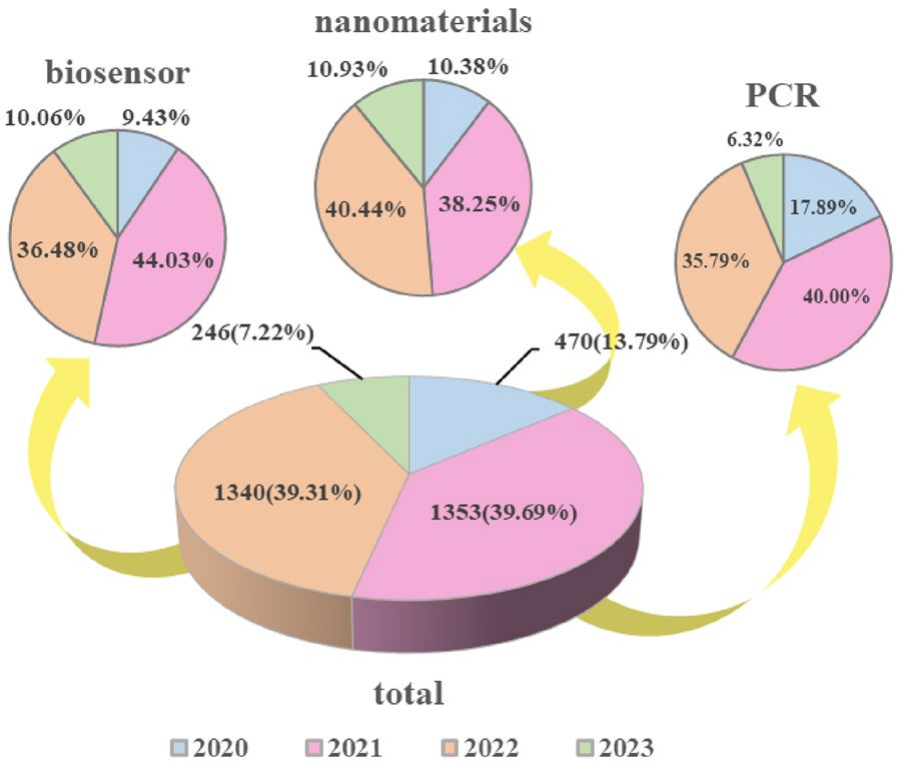
The pie chart of database “Web of Science” was used to analyze recent articles with the theme of rapid detection of COVID-19 and the search results with the keywords “biosensors”, “nanoparticles” and “PCR”

**Fig. 2:**
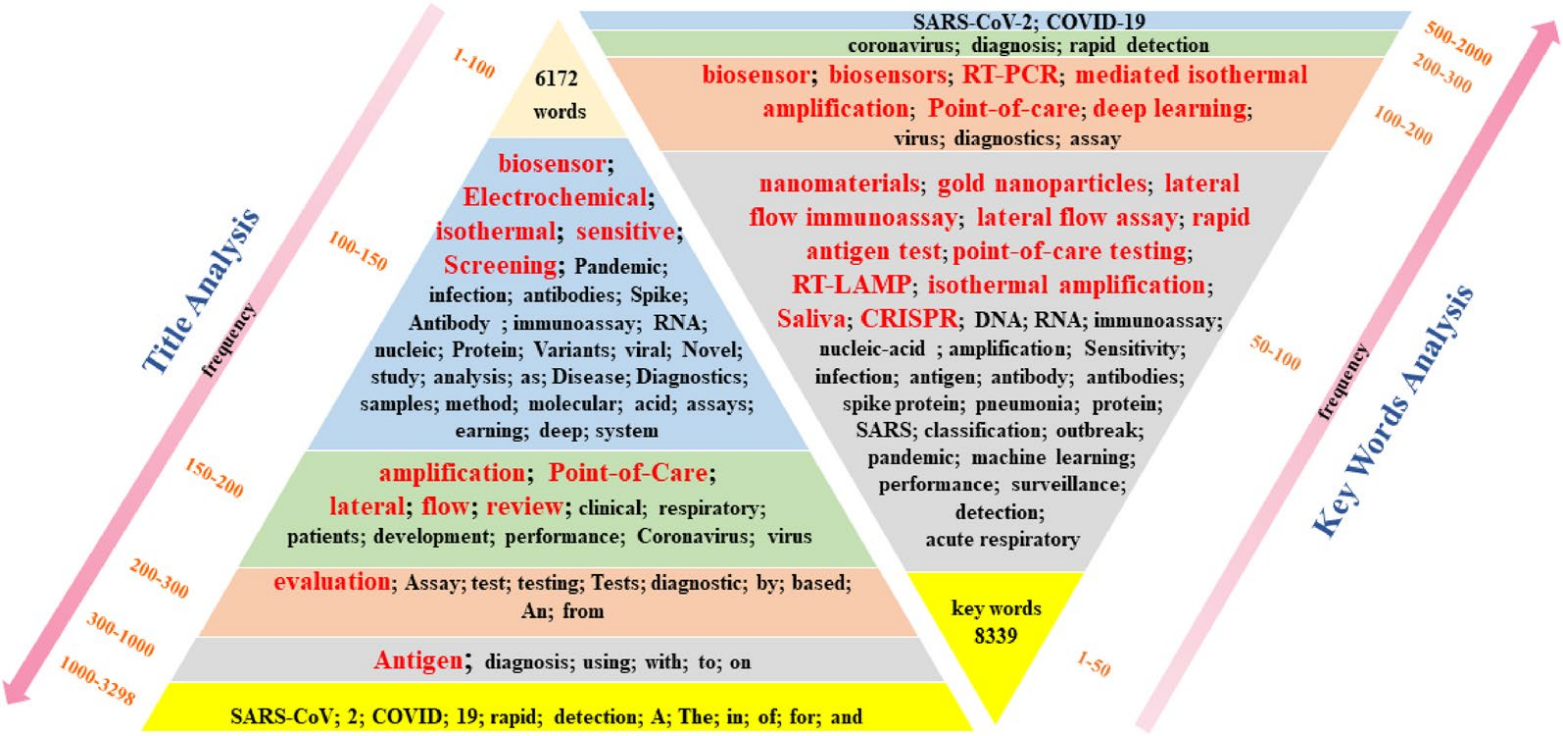
Title and keyword analysis of articles on the topic of rapid detection of COVID-19 in the database “Web of Science”

We conducted a biometric analysis of articles related to the rapid detection of COVID-19 since its emergence and searched for articles in the “Web of Science” database using the search formula “(TS = (COVID-19) OR TS = (SARS-CoV-2)) AND TS = (rapid detection)”. The result was 3566 articles, and 3409 articles were retrieved after searching directly for scientific papers. A statistical analysis based on the timing of these articles revealed (Fig. 1) that the number of articles published in 2020 due to the emergence of the COVID-19 shortly after was only 13.79% of the total, after which the amount of research exploded in 2021 (39.69%) and 2022 (39.31%). Since April of 2023, its relevant research reached only less than 1/5 (7.22%) of that in 2022. In addition, the titles and keywords of these articles were carefully analyzed (Fig. 2). When studying the words included in the titles, the words “antigen” and “evaluation” appear more frequently, followed by “amplification”, “Point-of-Care”, “lateral flow”, and “biosensor”, indicating the importance of Point-of-Care, nucleic acid amplification, lateral flow, and biosensor in rapid detection of COVID-19. Furthermore, the high frequency of the phrases “nanomaterials” and “gold nanoparticles” is eye-catching, probably due to the use of nanomaterials in rapid detection kits as well as biosensors. From the perspective of this review, nanomaterials are driving the development of rapid detection and their position in the field of detection is gaining ground with each passing day. The keywords “PCR”, “biosensor”, and “nanomaterials” were used and analyzed for all the retrieved articles. Figure 1 shows that the reports on “nanomaterials” had an increasing trend, unlike the other two keywords “biosensors” or “PCR”, indicating that nanomaterials with good performance are favored by more researchers.

**Fig. 3:**
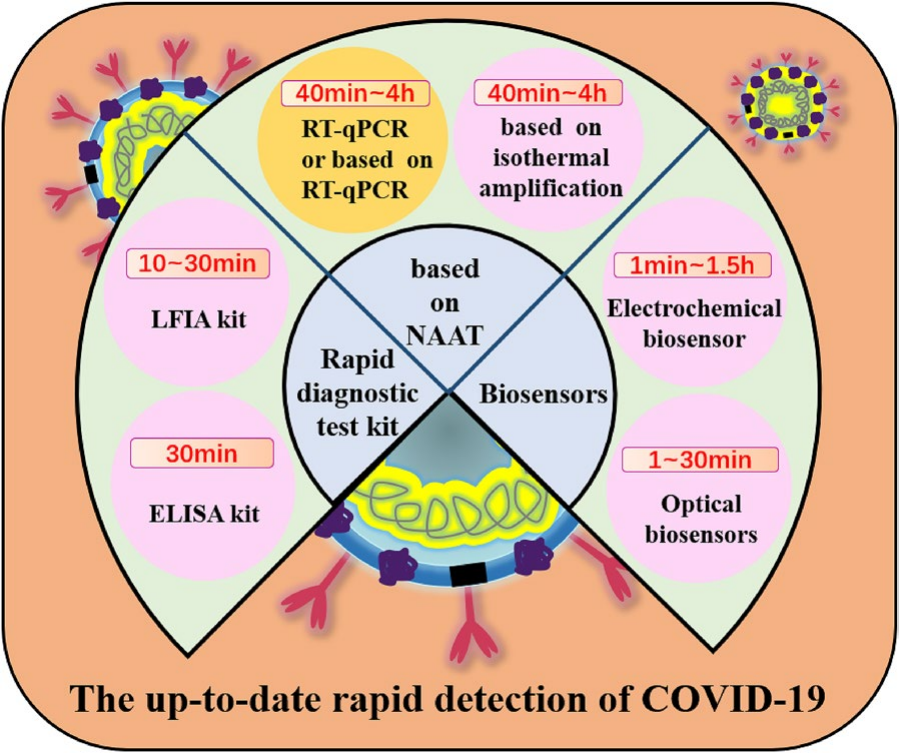
The types and methods of rapid detection of SARS-CoV-2 and the average detection time required for each method

Since the outbreak of COVID-19, many researchers have developed plenty of methods for rapid detection of SARS-CoV-2 and its variants, most of which rely on the development of nanotechnology that makes it possible to go beyond traditional RT-qPCR. In this article, we summarized recent reported methods so far for the rapid detection of SARS-CoV-2 based on nucleic acid amplification technology (NAAT) and lateral flow assay (LFA), and biosensor (Fig. 3). We compared these methods according to targets, testing principles and analytical performance, and provided an outlook on those methods for the rapid detection of SARS-CoV-2. The goal of this review is to explore recent rapid detection developments that are designed for specific detection of the full virus, viral protein, or antibodies against viral antigens from viruses. Given the large number of publications in this field, each section focuses on different techniques associated with rapid detection of specific viruses that mostly emerged in the last four years, especially related to SARS-CoV-2. This review will improve the management of the COVID-19 pandemic by encouraging people to self-quarantine, by preventing the spread of the virus, and by helping all prepare for future pandemics by allowing for faster response times.

## Testing principles

**Fig. 4:**
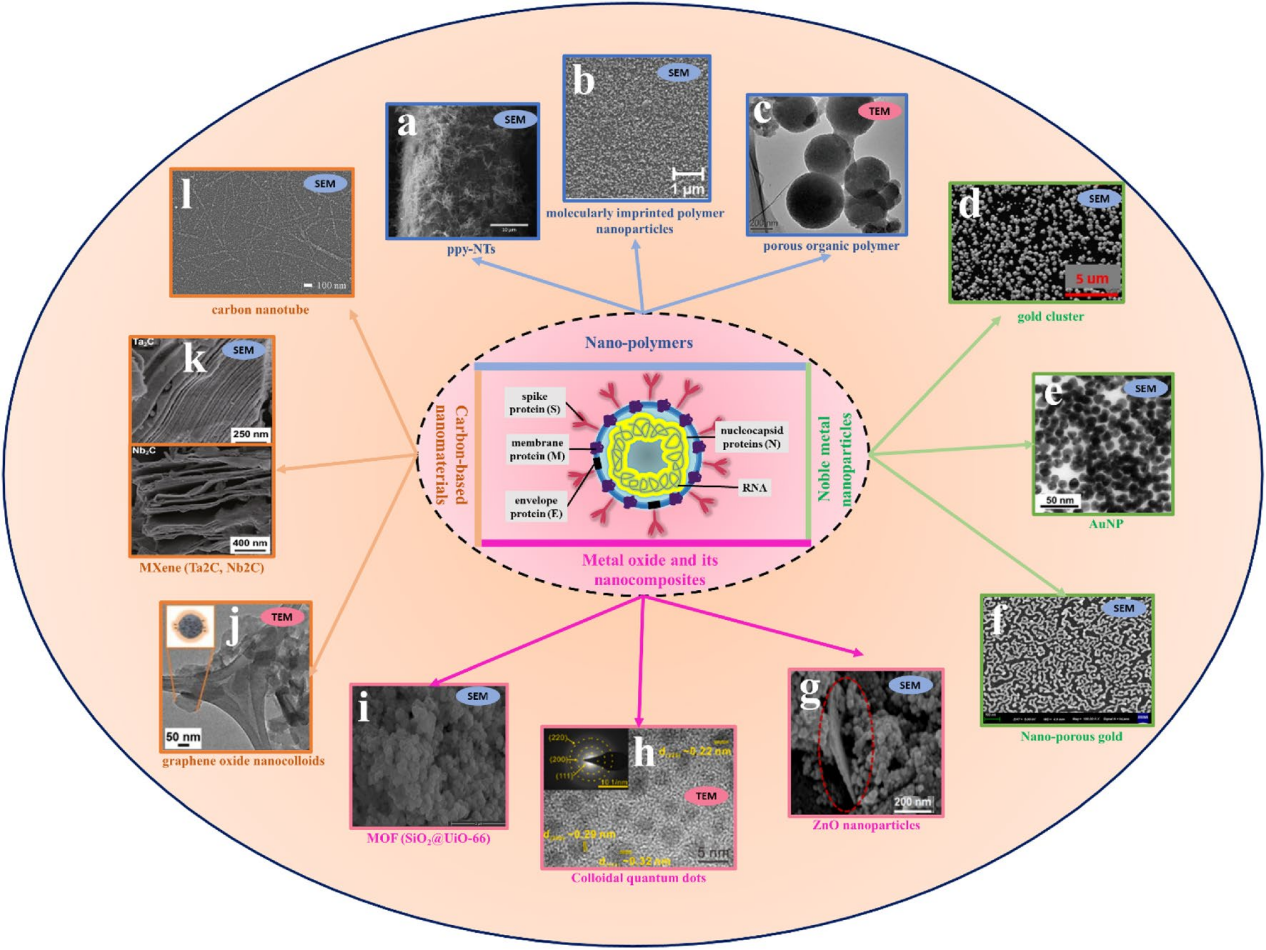
Biological structure and of the SARS-CoV-2 and enhanced biosensor by nanomaterials. **a** Reprinted with permission from ref. 47. Copyright 2022 Elsevier. **b** Reprinted with permission from ref. 48. Copyright 2022 American Chemical Society. **c** Reprinted with permission from ref. 49. Copyright 2022 Royal Society of Chemistry. **d** Reprinted with permission from ref. 50. Copyright 2022 John Wiley and Sons. **e** Reprinted with permission from ref. 51. Copyright 2022 American Chemical Society. **f** Reprinted with permission from ref. 52. Copyright 2022 Multidisciplinary Digital Publishing Institute. **g** Reprinted with permission from ref 53. Copyright 2022 American Chemical Society. **h** Reprinted with permission from ref 54. Copyright 2022 Elsevier. **i** Reprinted with permission from ref. 55. Copyright 2022 Elsevier. **j** Reprinted with permission from ref 56. Copyright 2022 Elsevier. **k** Reprinted with permission from ref 57. Copyright 2021 Springer Link. **l** Reprinted with permission from ref 58. Copyright 2022 Elsevier

Based on the biological structure of the SARS-CoV-2 (Fig. 4), there are three major methods to detect SARS-CoV-2: RNA, antigen (Ag) and antibody (Ab) [32]. Antigens include the spike protein (S), the envelope protein (E), the membrane protein (M), and the nucleoside protein (N). Methods that detect the RNA are mostly NAAT such as RT-qPCR and reverse transcriptase loop-mediated isothermal amplification (RT-LAMP), both of which have excellent sensitivity and selectivity. In comparison to RT-qPCR, RT-LAMP, proposed by many researchers, does not require use of expensive equipment and an RNA extraction step while reducing overall costs by speeding up the detection time in about 30–45 min [33–36]. However, RT-LAMP may produce false negatives due to improper sampling, transport, or handling. In addition, it may not be suitable for detection of mutated viruses. Notably, the false negative rate can be reduced by optimizing the NAAT process, such as adding a nucleic acid enrichment step, multiplex RT-qPCR, or creating a one-pot cyclic probe-mediated isothermal amplification protocol that combines the amplification and detection processes [37–40]. The rapid antigen test (RAT) targeting viral proteins has been shown to be used for the detection or monitoring of close contacts and high-risk groups with advantages of being easier, faster, and less costly, and disadvantages of being less sensitive than nucleic acid-based molecular tests [41, 42]. The sensitivity of RAT depends on the viral load of the sample based on data from a study suggesting that the sensitivity is only achieved when the viral load of the sample is high: the sensitivity is 90% for the cycle of quantification (Cq) range of 20–25 for RT-qPCR, and only 10% for the Cq range of 25–30 [43]. Abs test differs from other tests because it not only detects whether a person is infected but also reflects responses of the host after being vaccinated [32]. In summary, highly sensitive RNA assays and time-saving antigen assays are used to diagnose viruses, while Ab assays are used to aid in diagnosis and response to vaccine response.

Besides the above three principles for detection, there are other new but not mature testing strategies. For example, because patients infected with COVID-19 could exhale characteristic volatile organic compounds (VOCs), including 2,4-octadiene, 1-chloroheptane, nonanal(1a) and methylpent-2-enal (1b), a colorimetric method could be used to detect VOCs to determine the infection of COVID-19 with the advantages of being rapid, painless for asymptomatic infected patients [44]. Another way is to detect the main protease (M^pro^) because it is specific to SARS-CoV-2 during replication and transcription. Jin et al. [45] created a label-free peptide (ZY7) with a net neutral charge that could decompose into positively charged fragments in the presence of M^pro^, causing color changes in aggregation of negatively charged bis (psulfonatophenyl) phenylphosphine-modified gold nanoparticles (AuNPs), which is fast and convenient. Gut microbiota-Fusicatenibacter, as a very sensitive biomarker during SARS-CoV-2, may also become a new diagnostic tool. Hence, there is no relevant report available [46]. RNA, Ag, and Ab were used as target detectors in the method described in this paper.

## Rapid detection methods

### Methods based on NAAT

The gold standard method of NAATs, RT-qPCR, has evolved towards rapid, convenient, or simple techniques. Naranbat et al. [59] proposed a method characterized by the absence of viral (universal) transport medium and RNA extraction steps, which could greatly simplify the entire process such that test results could be available within only 1 to 2 h. Lee et al. [60] developed a deep learning model using the fluorescence values in each cycle of RT-qPCR, making sensitive predictions before the RT-qPCR results were available. Delpuech et al. [61] proposed to heat and inactivate SARS-CoV-2 samples prior to laboratory processing to reduce the overall cost, testing time, as well as safety hazard issues with less than 1 Cq loss in sensitivity compared to standard RT-qPCR. Chen et al. [62] developed a water-bath PCR that can quickly achieve thermal cycling and simultaneously detect SARS-CoV-2 with fluorescent LFA to make the whole process both faster and more sensitive. As an emerging detection technique, Digital PCR (dPCR) does not rely on a standard curve for the quantification of nucleic acid molecules and is highly sensitive for absolute quantification of RNA. It is even more reliable than RT-qPCR for the detection of SARS-CoV-2 in low viral load specimens or in wastewater [63–65]. Yolda-Carr et al. [66] developed a portable, real-time PCR device for the detection of SARS-CoV-2 in saliva samples, which consists of the SalivaDirect protocol [67] combined with the Ubiquitome Liberty16 system. This device could be connected to a smartphone to generate real-time test reports, which is more convenient, faster with improved sensitivity. For the detection of SARS-CoV-2 variants, one common method is to sequence the whole-genome. However, sequencing an entire genome requires relatively high costs. To address this problem, researchers [68, 69] established an RT-qPCR assay using the receptor-binding domain RNA of the spike protein of the SARS-CoV-2 variant as specific primers and probes. Xiong et al. [70] found two mutations, C1709A and C56G, that are specific to the genomes of Alpha and Delta variants. They established an amplification refractory mutation system combined with quantitative reverse transcription-qPCR based on these mutations, being able to complete full detection within 2.5 h. Dächert et al. [71] reported that the combination of variant‑specific PCR and nanopore-based full-length genome sequencing enabled not only rapid detection of the Omicron but also sensitive identification of newly emerging variants. Nucleic acid amplification on a chip is a highly viable potential technique for simplifying PT-qPCR while maintaining high sensitivity, which increases the possibility of rapid and accurate molecular diagnostics at home [72]. Another research work by Lee’s group [73] designed a multiplex RT-qPCR capable of simultaneously detecting SARS-CoV-2 and partial variants and integrated a microfluidic chip-based as a platform to reduce the detection time by more than half. For the RT-qPCR to be further improved, (1) optimization in the thermal cycling with precise temperature control and removal or reduction in the RNA extraction process are two important ways to shorten the overall detection time, (2) updates to the readout method is crucial to make RT-qPCR more portable, and (3) incorporation of multiplex RT-qPCR is an important means to detect mutant strains.

**Fig. 5:**
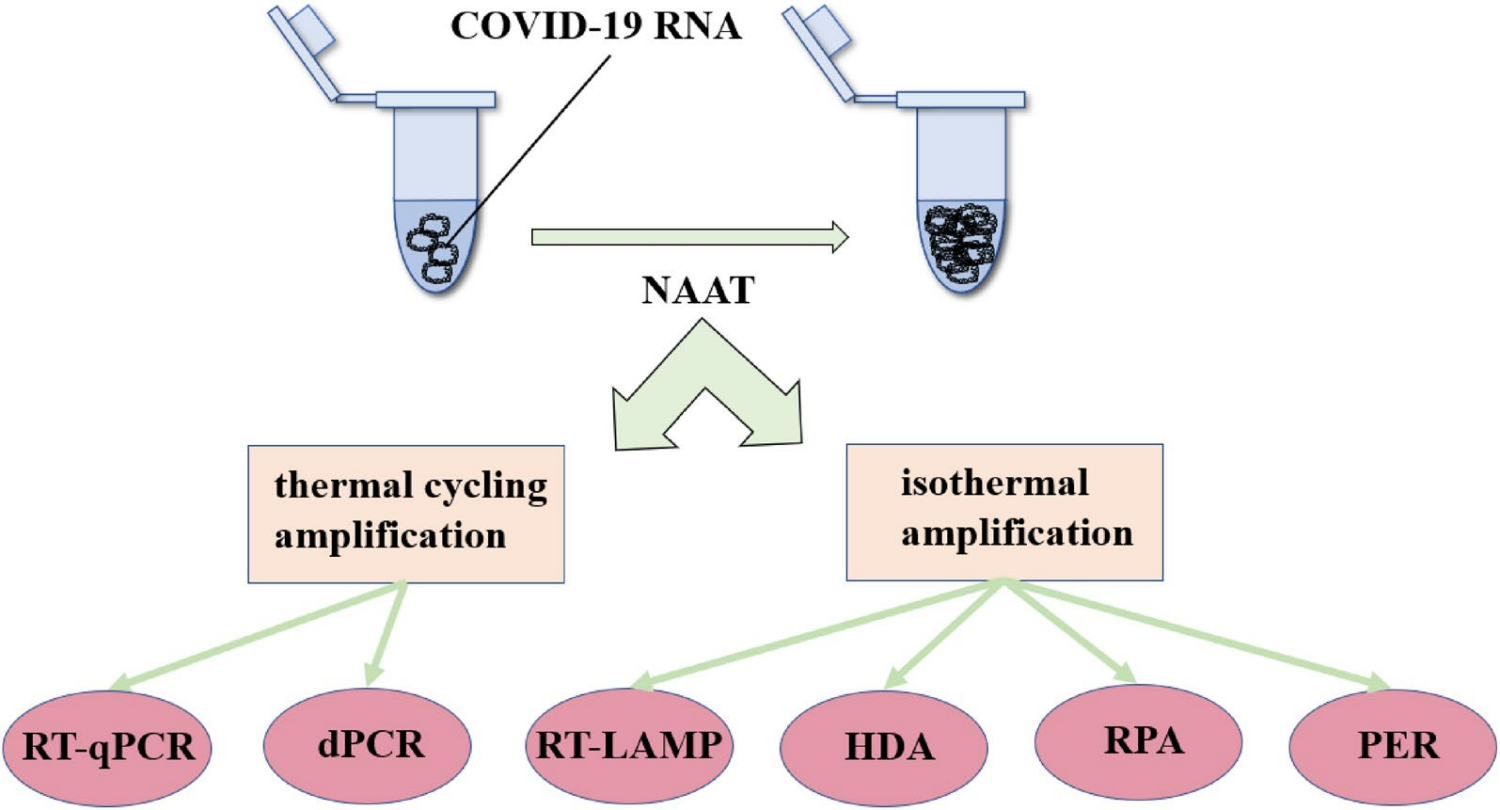
Different methods for nucleic acid amplification experiments

In addition to RT-qPCR, other methods of NAATs were also used for detection of SARS-CoV-2 (Fig. 5), among which RT-LAMP is widely used. Compared to RT-qPCR, the RT-LAMP assay process is faster in detection time, simpler in operation, and lower in overall cost. Many researchers have developed convenient and visualized assays that utilize RT-LAMP, making the whole process from sample to results less time-consuming. Several studies have reported LFA for RT-LAMP combined with CRISPR-Cas12 for SARS-CoV-2, which does not require thermocycling steps for amplification of the specific targeted nucleic acid while maintaining the selectivity and sensitivity levels [74, 75]. The advantage of this method is less time-consuming and could be visually detected by naked eyes. Colbert et al. [76] paired RT-LAMP with particle diffusometry, a particle imaging technique, to detect SARS-CoV-2, which means that just one smartphone device can be used for on-site testing. Iijima et al. [77] presented for the first time the detection of the L452R spike mutation by RT-LAMP coupled with a bioluminescent assay in real-time, which implies that RT-LAMP-based detection of mutant viruses is possible. In short, RT-LAMP for isothermal amplification of nucleic acids greatly compensates for the time-consuming problem of RT-qPCR.

The development of isothermal amplification technology has diversified the methods for NAAT-based detection of RNA. Shanmugakani and Wu [78] developed a reverse transcription helicase-dependent amplification (RT-HDA)-coupled dipstick technique, which does not require thermal cycling or expensive equipment while saves time. Researchers also reported detection of SARS-CoV-2 based on a rapidly integrated recombinase polymerase amplification (RPA), which is a novel isothermal amplification technique to complete amplification in 15–20 min [79–81]. Li et al. [82] used primer exchange reaction (PER) to amplify nucleic acids, which was combined with CRISPR-Cas12 for rapid detection of SARS-CoV-2. Since PER is performed by automatic extension of short primers to sequence-specific single-stranded DNA after a target-catalyzed hairpin template in the presence of a strand displacing polymerase, it is faster and easier than reverse transcription-mediated amplification. However, expensive and heavyweight equipment on nucleic acid amplification is still a common problem for NAAT assays.

### Rapid diagnostic test kit

#### Enzyme-linked immunosorbent assay

Enzyme-linked immunosorbent assay (ELISA) is often used for the detection of viral antibodies and has been developed as a rapid diagnostic test kit due to its ease of operation and use of inexpensive equipment. In ELISA of SARS-CoV-2, different structural proteins could be used as Ag to detect the corresponding antibodies and researchers have developed ELISAs with good sensitivity and specificity [83–86]. Using microfluidic technology, González-González et al. [87] developed an automated ELISA chip for detecting antibodies to SARS-CoV-2, enabling on-site testing that may only require a smartphone with a camera. Kasetsirikul et al. [88] invented a paper-based ELISA for detection of antibodies to SARS-CoV-2, which could significantly reduce costs and make the test faster than conventional ELISAs. It could be completed within 30 min. Due to the increase in vaccination and cured patients, the SARS-CoV-2 Ab test cannot be used as a diagnostic tool but only as a diagnostic aid or a way for post-vaccination evaluation. Therefore, researchers have developed ELISA-based RAT. Domenico et al. [89] prepared a rapid test kit for simultaneous detection of two antigens using a double antibody sandwich method, which has the advantage of being fast at about 30 min, simple, and directly observable with the naked eye, but it could not detect Ag at low concentrations. To some extent, ELISA is able to characterize viruses in a more time-efficient and portable way than the gold standard while it might not specifically detect the RNA of viruses.

#### Lateral flow immunoassay

**Fig. 6:**
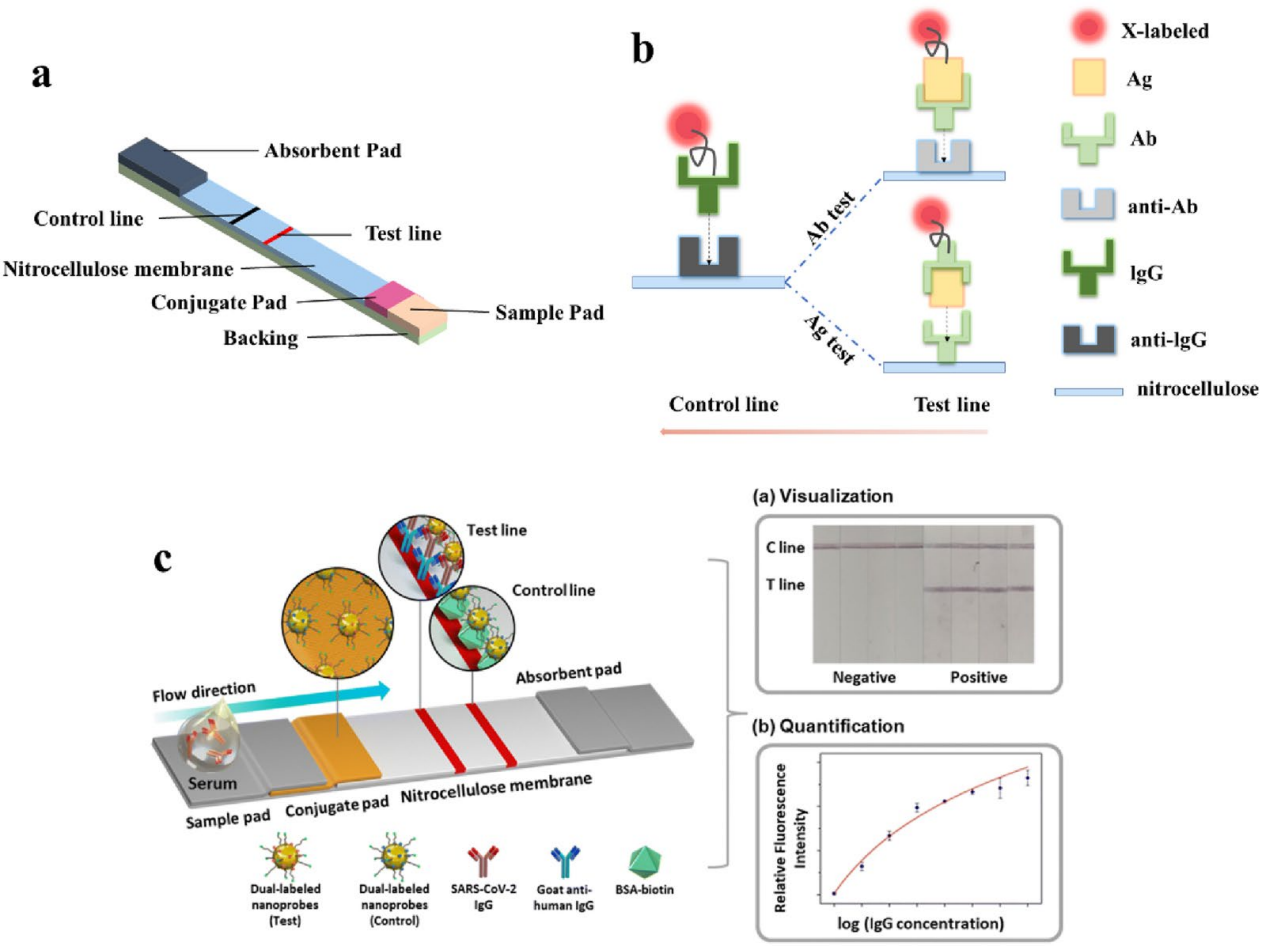
Schematics of a typical LIFA for COVID-19 diagnostics. **a** Components of a LIFA test kit. **b** Detection principles of Ag test and Ab tests. **c** Mechanisms for the functioning of LFIA. Reprinted with permission from ref 181. Copyright 2022 Royal Society of Chemistry

Compared to ELISA, lateral flow immunoassay (LFIA) is more stable because of labels, such as AuNPs and fluorescein isothiocyanate on a paper-based diagnostic platform, which makes it more suitable for commercialization. The utilization of nanoparticles as labels has gained attention in developing rapid diagnostic test kits for improved diagnosis and treatment. The conventional LFIA device are generally composed of three major parts, i.e., substrate based on papers, antibodies or antigens as detection element and reporters as signal-transforming element (Fig. 6). The fabricated structure, principle and detection mechanism of LFIA are also shown in Fig. 6. The structure of LFIA generally consists of sample pad, conjugated pad, test pad, absorbent pad and backing pad. When an assay is carried out on a LFIA, a small volume of sample is dropped onto the pad, migrates on the conjugated pad, then carries conjugated particles to the test pad. Target such as in the given sample are recognized and bonded with detection antibodies on reporter surface in conjugated pad, where complexes interact with capture antibodies on test line and free reporters bound on control line. LFIA with flow-through immunoreactivity on a Nitrocellulose membrane specifically recognizes SARS-CoV-2 antigens and antibodies and produces an optical signal visible to the naked eye. The control line is designed to improve the specificity of the assay and thus avoid false negatives.

The key issue of LFIA is the relatively lower detection sensitivity and efficiency that still needs further improvement. Notably, nanomaterials are a decisive factor and a significant contributor to improve the performance of COVID-19 rapid diagnostic kits [90]. Peng et al. [91] enhanced the sensitivity by depositing copper on AuNPs-labeled LFIA test papers, resulting in a detection limit of 10 pg/mL for this RAT. Szekely et al. [92] combined carboxy gold nanoshells with antibodies to form stable conjugates to target low mutation rate ‘N’ to obtain a rapid diagnostic test kit with sensitivity comparable to RT-PCR. Lee et al. [93] developed an LFIA-based sandwich immunoassay for determination of antibodies to SARS-CoV-2, using colored cellulose nanobeads to label secondary antibodies in the sandwich structure to reflect the presence of antibodies to neo-coronavirus. Chen et al. [94] successfully labeled ‘N’ with selenium nanoparticles and developed a rapid LFIA-based test for detecting antibodies to SARS-CoV-2 with results readable within 10 min. Zhang et al. [95] reported two highly sensitive LFIAs for the detection of SARS-CoV-2 receptor binding domain (RBD) and ‘N’ using AIE luminophores with good optical properties and less susceptibility as a fluorescent label. Duan et al. [96] applied ratiometric fluorescent analysis for dual-detection LFIA for the first time, using carboxyl-functionalized Europium chelate nanoparticles to label the RBD and set up human angiotensin-converting enzyme 2 (hACE2), which can bind to RBD as a calibration line. Their results showed higher precision and sensitivity with a wider dynamic linear range for Ab detection. Dighe et al. [97] used antisense oligonucleotides labeled with 6-carboxyfluorescein and biotin to specifically identify SARS-CoV-2 genes as probes using AuNPs capped with cysteamine as control signals to improve the sensitivity of this LFA-based detection of SARS-CoV-2 RNA. The possible downside of this approach is lower sensitivity and false positives [98–100]. Therefore, a top priority is to develop highly sensitive LFIA-based rapid detection kits. LFIA test strips are considered the economical alternative for the instant diagnosis of COVID-19 in public health centre [198]. The compositions and properties of these components are closely related to the performance of paper-based POC immunoassays. (e.g., traditional paper and emerging paper materials) and principles (e.g., interface). More research is focused on promoting high-throughput immune-analyzers for mass screening. The sensing principle involves detecting analytes (could be an Ag or Ab) with the help of secondary antibodies conjugated with labels such as gold nanoparticles, fluorescent molecules and quantum dots, promoting visual sensing of color changes.

The key issue of LFIA is the relatively lower detection sensitivity and efficiency that still needs further improvement. The biosensor for SARS-CoV-2 virus or other targets based on LFIA with lower prices, better stability and lower detection limit. There is no doubt about LFIA will bring a huge effect to the present POCT market.

LFIA-based rapid diagnostic test kits are now commercially available with the form of rapid diagnostic kits on the market, most of which are LFIA-based RATs. Compared to RATs, rapid antibody test kits are inferior as a diagnostic tool but they could be used as a screening tool [101, 102]. Some investigators evaluated the limits of detection (LOD) of different kits and showed that most of them are sensitive to detect SARS-CoV-2 [103–106]. Many researchers have compared and evaluated these kits with PCR and found that almost all kits on the market have 100% specificity, but sensitivity varies widely, with only a very few having relatively higher sensitivity than others [107]. The sensitivity of RAT highly depends on the viral load and high viral loads leading a sensitivity of over 90% for RAT [108]. Table 1 indicates that several antigen detection kits are currently available on the market: STANDARD Q COVID-19 Ag tests (SD Biosensor Inc., Korea), Panbio^™^ COVID-19 Ag Rapid Test Device (Abbott, Germany), etc. Table 1 also shows that the target of the RATs is ‘N’ and the detection time is mostly around 15 min. It has high sensitivity and meets WHO requirements (at least 80% sensitivity and 97% specificity compared to RT-qPCR) in symptomatic patients or exposed individuals. However, for asymptomatic populations or areas with low infection rates, the sensitivity is not comparable to that of RT-qPCR [109]. It is worth noting that although RAT sensitivity is lower compared to RT-qPCR, the use of RAT will reduce much of the burden. In the near future, RAT may shorten the isolation time in the face of a huge number of people needs to be tested [110, 111]. The collection of samples from different sites (nasopharyngeal swabs, nasal swabs, and saliva) also had a greater effect on sensitivity, with nasopharyngeal swabs having the best sensitivity and saliva having the worst sensitivity. Even though the sensitivity of saliva is poor, it can reduce the pain of the subject during sampling, so it makes sense to develop a highly sensitive saliva test kit. In addition, combinatorial antigen test kits enable the combination of two antigen test kits, which have been shown to increase sensitivity [112]. The sensitivity of the available RATs has been reduced for mutant strains, but they are still an irreplaceable screening tool because of their high efficiency [113]. In general, commercially available RATs are highly variable for various reasons including viral load, variant strains, target population, etc. despite their outstanding high efficiency.

**Table 1:**
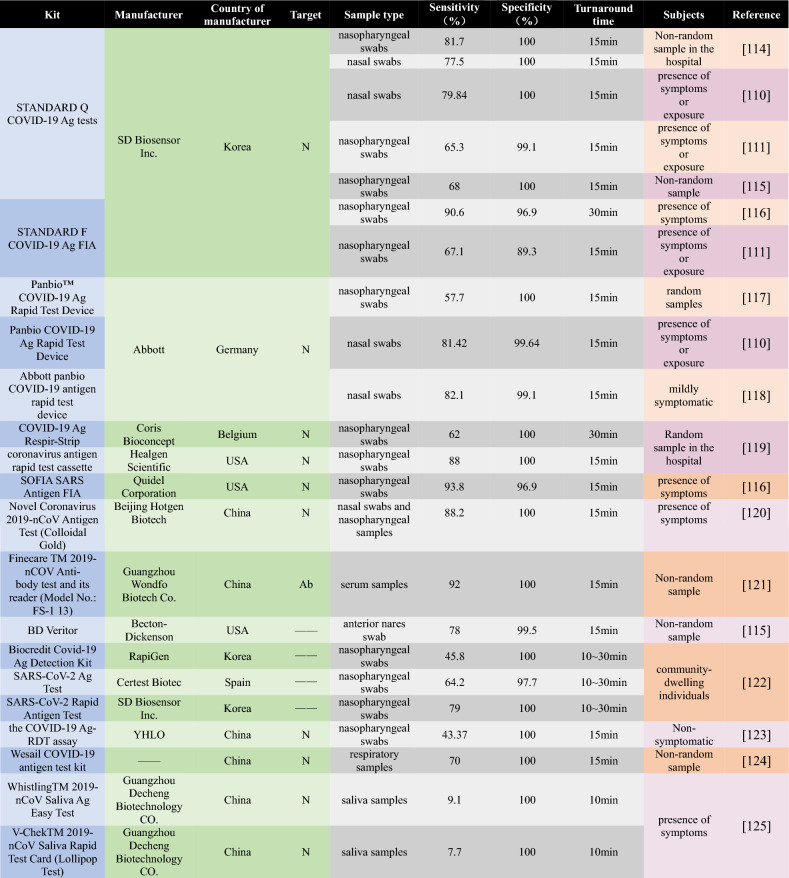
Evaluation data against commercially available LIFA rapid test kit

In the critical context LFIA has been commonly used for POCT of SARS-CoV-2 due to its low cost and portability, which could be an instrumental factor in the successful fight against the outbreak [182]. Although LFIA-based rapid test kits can fulfill the criteria for POCT, it still confronts some challenges such as improved sensitivity, poor stability and inability to detect nucleic acids [29]. Similarly, NAAT-based SARS-CoV-2 POCT tends to be used in healthcare settings rather than for self-testing [183]. Accordingly, novel ideas for better use in SARS-CoV-2 POCT have been proposed. For example, Ran Liu et al. [184] combined a CRISPR-Cas12-based assay for nucleic acids and a portable meter, and finally have refined the detection of N in the system, which enables patients to quantitatively test for a wide range of SARS-CoV-2 markers at home with a portable device. It is worth noting that SARS-CoV-2 POCT, centered on biosensing that is free from laboratory dependence, is being developed with great enthusiasm and has the potential to be generalized in the future. The structural design using biosensing and the introduction of smartphones can satisfy the demands of POCT even more, but its commercialization needs to take into account cost, biosafety, data security, and stability [185]. In addition, microfluidic chip-based POCT for SARS-CoV-2 is considered an ideal diagnostic tool for pandemic response. Its small dimensions and portability, high detection efficiency and considerable commercialization value have increased its popularity, which has led to the development of a variety of microfluidic platform-based designs and assays including nucleic acid amplification, immunosensors and biosensors [186–188]. Overall, POCT has significant implications for pandemics similar to SARS-CoV-2, and despite the development of multiple portable devices for POCT in addition to LFIA, there are serious challenges in actual commercial development.

### Biosensors

#### Electrochemical biosensor

**Fig. 7:**
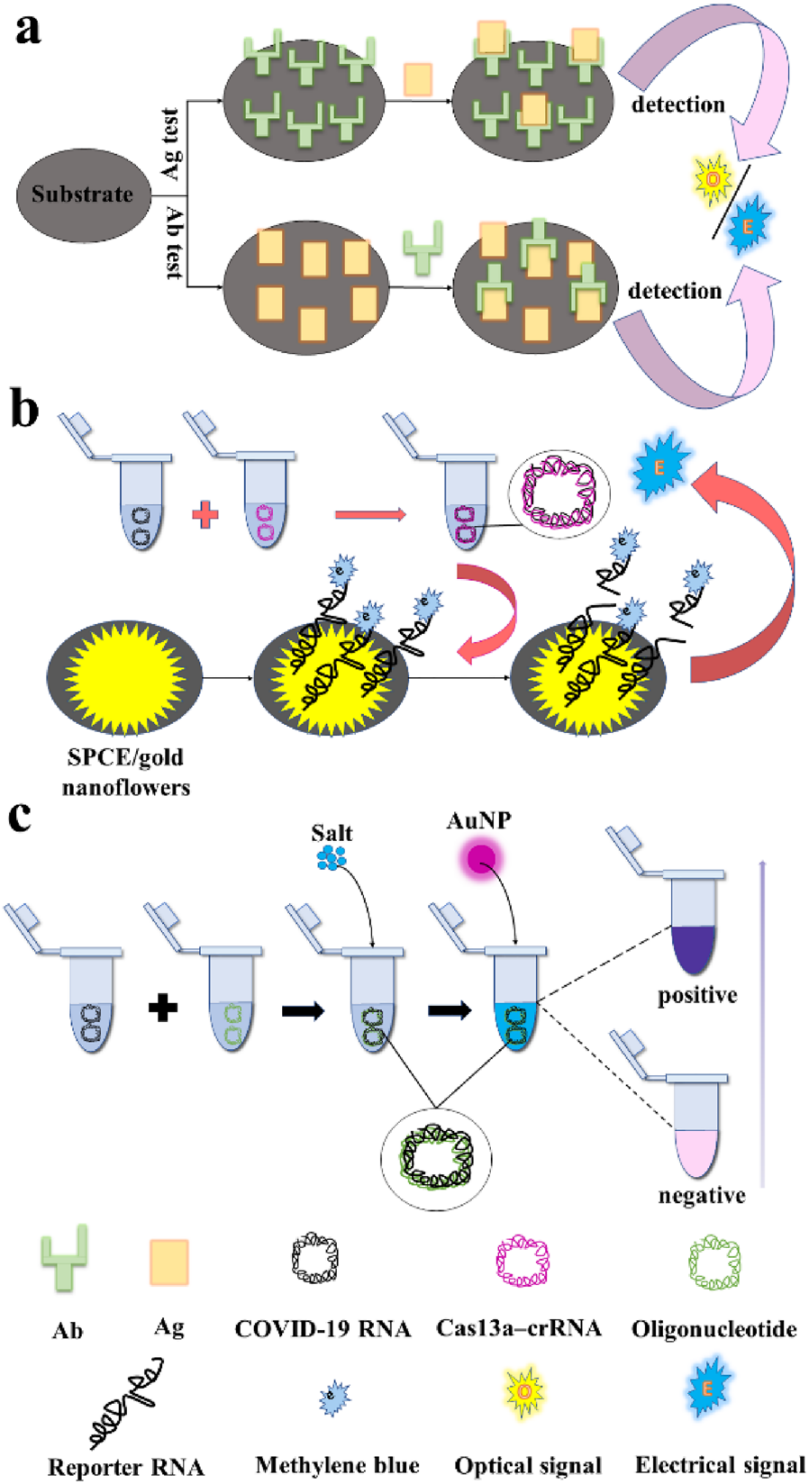
Biosensor platforms for detection of SARS-CoV-2. **a** Biosensors for Ag and Ab detection. **b** One electrochemical biosensor for RNA detection. **c** One colorimetric biosensor for RNA detection

Electrochemical biosensors are capable of rapidly converting biological signals into electrical signals. They can provide enhanced selectivity and sensitivity and are widely used in virus detection because of shorter reaction times and use of less sample volume. Even so, there is still a huge challenge regarding its signal amplification, stability, and commercialization [26, 126]. Among the electrochemical biosensors to detect SARS-CoV-2, immunoimpedance biosensors for Ag and Ab detection are preferred (Fig. 7a). Its sensitivity could be enhanced by modifying the electrodes using nanoscale materials with good conductivity such as AuNPs and single-walled carbon nanotubes [127, 128]. Screen-printed carbon electrode (SPCE)-based biosensors are compact, fast, and low cost with the value of potential commercialization [129, 130]. Haghayegh et al. [53] modified buffer-based zinc oxide/reduced graphene oxide on the SPCE surface to increase the electrical signal, and this RAT was able to detect ‘N’ within 15 min. Soto and Orozco [131] developed an immunoimpedance biosensor by modifying functionalized processed peptides capable of specifically recognizing ‘S’ on screen-printed gold electrodes. Polypyrrole is favored by researchers for its better surface area, high electrical conductivity and electrochemical activity, and its synthesized nanotubular form with better properties than the spherical form as a substrate [47]. Mehmandoust et al. [55] synthesized SiO_2_@UiO-66 nanocomposite as a metal–organic framework and modified it on SPCE to greatly improve the conductivity of the electrode, which is capable of sensitively detecting SARS-CoV-2 Ag. The road to commercialization is not far away with the increasing development of screen-printed, electrode-based impedance immunosensors.

For current and voltage biosensors, biosensor sensitivity and convenience can also be improved by modifying materials with excellent properties or selecting materials with better structural properties as substrates. Zhao et al. [54] modified colloidal quantum dots with increased surface area and dangling bonds on the electrode to firmly adsorb the SARS-CoV-2 Ag, and the electrochemical biosensor was able to detect SARS-CoV-2 antibodies in less than one minute. Liv and Kayaba [50] prepared a gold cluster and Ag modified on a glassy carbon electrode (GCE) for the detection of antibodies to SARS-CoV-2. Kim et al. [132] developed a RAT with a LOD of 1.17 fg/mL by immobilizing an Ab on a dual-gate oxide semiconductor thin-film transistor that amplifies electrical signals as a substrate. Whether voltage biosensors, current biosensors or impedance biosensors, most of them use antigen–antibody specific binding to detect Ag or Ab.

However, the electrochemical biosensor-based approach to detect SARS-CoV-2 is not limited to Ag and Ab detection and can directly detect RNA without relying on NAAT. Heo et al. [133] immobilized the reporter RNA labeled with methylene blue and biotin labeled ends on a SPCE modified with nanocomposites and gold nanoflowers to form an electrochemical aptamer biosensor for the detection of SARS-CoV-2 RNA (Fig. 7b). The biosensor incorporated CRISPR-Cas13 and formed a complex of the target RNA with Cas13a-crRNA, which cleaved the reporter RNA immobilized on the electrode to produce a change in the electrical signal.

To date, an increasing number of electrochemical biosensors emerged for the detection of SARS-CoV-2. Park et al. [134] applied multiple vertically paired electrodes to develop a capacitive biosensor for detection of SARS-CoV-2 Ag with higher sensitivity than conventional capacitive biosensors based on interdigitated electrode. Jiang et al. [135] modified a magnetic capture probe on a screen-printed gold electrode and then hybridized Ru(bpy)_3_^2+^-labeled signal probe with electrochemiluminescence (ECL) signal to the SARS-CoV-2 RNA. The obtained biosensor could specifically identify the RNA and generate a highly sensitive ECL signal with the detection range of 0.1 fM to 10 µM. McClements et al. [48] used molecularly imprinted polymer nanoparticles to create an imprint of SARS-CoV-2 Ag and modified it on a screen-printed electrode to detect the Ag, a molecularly imprinted biosensor that is more stable and reliable and can deliver results within 15 min. Yet these biosensors were apparently developed without attention to their commercialization possibilities despite their outstanding sensitivity and innovation.

Field-effect transistor-based biosensors (BioFETs) are highly sensitive, have a wide detection range, and can be made ultra-sensitive with high electron mobility transistors. However, they are often limited by their high cost, poor reproducibility, and lack of portability [136–138]. Researchers have combined BioFET with enzymatic reactions to collect electrical signals generated by changes in pH of the solution to detect SARS-CoV-2 Ag or antibodies, and have found that the application of phosphatase is more stable than the application of urease [139]. Chen et al. [140] have developed a portable biosensor for in situ detection of N in saliva based on an electrical double-layer gated BioFET system, which can be read on an iPhone through a portable reader. Electromechanical biosensors formed by the combination of microelectromechanical systems and field-effect transistors have ultra-high sensitivity. Researchers have developed BioFET for the detection of SARS-CoV-2 RNA using carbon nanotubes as the substrate and RNA hybridization as the signal generator [141]. Wang et al. [142] created an ultra-fast and portable electromechanical aptamer biosensor for ultra-fast detection of SARS-CoV-2 RNA that does not require nucleic acid amplification by using flexible single-stranded DNA linked by rigid tetrahedral double-stranded DNA as a probe. Because of its important entry level among all electrochemical biosensors, development of portable and low-cost commercially available BioFETs have attracted a lot of researchers.

#### Optical biosensors

The optical biosensors currently used for rapid detection of SARS-CoV-2 are colorimetric and immunofluorescent biosensors as well as biosensors based on spectroscopic techniques. The biggest advantage of colorimetric and immunofluorescence biosensors is that the results are usually visible to the naked eye (Fig. 7c). Mohamad Mahani et al. [143] reported the FRET-based aptasensor for interleukin-6 as a biomarker for COVID-19 progression using nitrogen-doped carbon quantum dots and gold nanoparticles. Alhadrami et al. [144] reported a colorimetric biosensor of using a cotton swab as a substrate to collect the detected S through the lactoferrin general capture agent, it could specifically bind an orange nanopolymer-modified Ab to produce an optical signature visible to the naked eye, which is available in 5 min and suitable for field detection. The label-free detection of SARS-CoV-2 spike protein is demonstrated by using slightly tapered no-core fiber (ST-NCF) functionalized with ACE2.The ACE2-immobilized ST-NCF sensor head was exposed to the samples of SARS-CoV-2 spike protein with concentrations ranging from 1 to 104 ng/mL [145]. Kang et al. [146] developed a hairpin structure of hACE2 mimetic peptide beacon, which has only a weak fluorescence signal due to the fluorescence resonance energy transfer effect in the normal state, and the hairpin structure is opened to generate a fluorescence signal when affected by S. The whole process could be completed within 3 h. These two types of biosensors are usually combined with LFIA to form the rapid detection kits mentioned in this paper. Feng Long constructed a new all-fiber Fresnel reflection microfluidic biosensor which was constructed through combining all-fiber optical system, microfluidic chip, and multimode fiber bio-probe. The limits of detection of SARS-CoV-2IgM and SARS-CoV-2 IgG were 0.82 ng/mL and 0.45 ng/mL, respectively [147].

The use of spectroscopic techniques such as dynamic light scattering and surface-enhanced Raman scattering (SERS), which are faster, more economical, and more accurate than traditional detection methods, for the detection of SARS-CoV-2 is just around the corner [148–150]. Kawasaki et al. [151] used an imprinted photonic crystal film as a substrate and immobilized antibodies on its surface to identify Ag with high sensitivity, and finally performed simple reflectance measurements by an optical device equipped with a spectrometer in a smartphone. Hadi et al. [152] combined U-Bent plastic optical fiber with nanogold to immobilize the Ab and used it as a probe to build a fiber optic biosensor, and finally diagnosed the presence of N by the change of the biosensor optical intensity. Optical biosensors based on surface plasmon resonance are increasingly used for the detection of new coronaviruses due to their high sensitivity [153]. Rahmati et al. [154] reported a new detection strategy which was used to improve the sensitivity of SARS-CoV-2 spike receptor-binding domain based on a lateral flow immunoassay platform utilizing a delayed hydrophobic barrier fabricated. Zheng et al. [52] developed a localized surface plasmon resonance (LSPR) biosensor based on a vertical microcavity with nano-porous gold modified on its surface and immobilized with antibodies to the SARS-CoV-2, which generates an optical signal when the target Ag is captured by the biosensor. Liang et al. [155] combined the LSPR biosensor with optical imaging and artificial intelligence methods to be able to detect new coronaviruses within 12 min. These types of optical biosensors are moving towards portability and commercialization because of their high sensitivity, possibility to save time, and low cost compared to electrochemical biosensors.

#### Nanomaterials for biosensors

##### Variety of nanomaterials

Biosensors tremendously advanced for the detection of virus, pathogens and microorganisms [156]. Nanomaterials commonly used in the fabrication of biosensors to improve their performance [157], where they can improve the true positive rate of biosensors and in the green synthesis to make biosensors sustainable and environmentally compatible [158, 159]. Figure 4 shows various nanomaterials used as biosensors for the detection of SARS-CoV-2. Noble metal and inorganic metal oxides nanoparticles assume an important role in improving sensitivity and accuracy, such as nano-gold, nano-silver, and nano-zinc oxide [160–162], but bimetallic nanomaterials can greatly increase the performance of biosensors compared to the monometallic as excellent signal amplifiers, and some are even called nanozyme because of their catalytic properties [163]. The stability and high activity of such nanozyme also make it convenient to store them for a long time, which makes it possible to use them as specific identifiers for the preparation of biosensors [164]. Nevertheless, low biocompatibility is a fatal drawback for metallic materials, the use of carbon-based materials can overcome this limitation and it has been shown that the use of carbon materials as substrates is a potential area of rapid development for biosensors [160, 165, 166].

When it comes to carbon-based materials, carbon nanotubes with high specific surface area, good electrical and thermal conductivity have been widely used for biosensors [167], especially, graphene nanomaterials with excellent biocompatibility have become a new buzz with vast attention, and graphene-based electrochemical biosensors with ultra-precise detection capabilities have been heavily worked on [168–172]. Graphene and its derivatives are undoubtedly desirable materials for the construction of efficient virus detection biosensors. Wei Li Ang et al. [56] discussed the use of graphene nanocolloids as electroactive materials to develop an electrochemical biosensor for the detection of SARS-CoV-2 RNA in the range of 10^–10^ M to 10^–5^ M. Furthermore, MXenes with unique two-dimensional structure and good electrical conductivity and ductility, have become attractive materials in the development of biosensors, but they have synthetic material waste disposal and mass production stability problems still to be solved [173, 174].

##### Application of nanomaterials

Nanomaterials are applied in the diagnosis of COVID-19 through portable colorimetric devices and biosensors with various transduction mechanisms. AuNPs and its complexes have been widely used in rapid test kits and colorimetric sensors to make the test results visible to the naked eye. Precious metals, carbon-based nanomaterials and conductive polymers with favorable electrical conductivity as well as the limitations and accuracy of detection enhance the electrochemical activity of electrochemical sensors such as voltammetric and impedance biosensors [201]. For optical sensors such as FRET, SERS and LSPR sensors nanomaterials with excellent optical properties have great applications [52, 143, 148]. Besides, nanomaterials have been continuously utilized in sensors such as ECL sensors, FET sensors, and so on [203]. Notably, magnetic nanomaterials were successfully applied to amplifier of genome. S B. Somvanshi et al. [206] report the fabrication and application of surface-functionalized magnetic zinc ferrate nanoparticles for the rapid detection of SARS-CoV-2 RNA, and the proposed model allows RNA extraction from multiple samples. It has to be recognized that nanomaterials are being applied to a wider category of biosensors.

##### Function of nanomaterials

Five kinds of nanomaterials including nanoparticles, nanowires and nanorods, carbon nanotubes, and quantum dots have been currently well utilized in biosensors to improve sensing efficiency and diagnostic sensitivity [167]. Nanomaterials in biosensors mainly take advantage of structural, conductive, and optical properties to obtain a larger specific surface area as well as to increase the rate of electron transfer, similarly, nanoparticles such as AuNPs are often used as signal transducers or nano-lanterns to become an important component in electrochemical and optical sensors [189–192].

With the advancement of nanotechnology, more and more functions of nanomaterials are being developed for the construction of biosensors and play an even more irreplaceable function in the diagnosis of COVID-19. Nanomaterials with excellent construction can contain and modify biomolecules in a superior manner. Nanomembrane graphene was synthesized by binding AuNPs and nano-islands on reduced graphene oxide, which is capable of remarkable binding of S and Ab with an affinity constant of 0.93 × 109 M^−1^ [193]. Andrei Pligovka et al. synthesized complexly structured two-level 3D cylindrical nanomembranes by stepwise oxidation, and the optical properties of this material may have great potential for application in label-free optical biosensors [194]. Secondly, nanomaterials possessing specificity can serve as receptors instead of less stable biomolecules as core members of the sensing mechanism. Biosensors based on biomimetic nanomaterials as well as molecularly imprinted nano-polymers as recognition systems for the detection of SARS-CoV-2 have been developed, which opens a new chapter in the integration of nanomaterials into biosensors [195, 196]. In addition, nanofiber membranes fabricated based on electrostatic textile technology are fine flexible substrates for wearable sensors for diagnosis of COVID-19 [197]. Altogether, nanomaterials in future research may act as substrates, receptors, signal transducers and powerful bio-binders thus becoming an integral part of biosensors.

## Conclusion and future perspectives

To date, methods for rapid detection of SARS-CoV-2 fall into three broad categories: NAATs, rapid diagnostic test kits, and biosensors. NAAT based methods were mainly used to detect SARS-CoV-2 RNA by reducing the required time to improve PCR or using more rapid nucleic acid amplification techniques such as LAMP, RT-HDA, and RPA. There are two types of rapid diagnostic test kits, one that utilizes ELISA (which usually requires labeling with enzymes) and another that uses LFIA (which tends to use more stable chemical labels). While ELISA is only suitable for detection of SARS-CoV-2 antigens and antibodies, LFIA is dominated by RAT although it is also proposed for RNA detection. For detection of variants, in contrast, researchers tend to use less mutable N as the target of RAT thus avoiding false negatives. Biosensors are the preferred detection platform for researchers due to their good sensitivity and selectivity as well as the short detection period. They could be divided into electrochemical biosensors and optical biosensors depending on the output signal. Their targets could be Ag, antibodies, and RNA. With the continuous development of biosensors, a variety of biosensor platforms have emerged for the detection of SARS-CoV-2, among which BioFET, screen-printed sensors, and optical biosensors based on SPR are more favorable and generally used.

The performance of biosensors could be affected by different sensing methods and modification materials. First of all, receptors such as antigens, antibodies, aptamers, and molecularly imprinted polymers as recognition systems targeting SARS-CoV-2 play a decisive role in the performance of the sensors. Although immune response-based sensors have been developed and are well established for the diagnosis of COVID-19, the inability to detect RNA as well as poorly stabilized antigens and antibodies has been a hindrance to development. In contrast, more stable aptamer sensors are robust in detecting different types of SARS-CoV-2 biomarkers (RNA and Ag) and have excellent specificity to reduce false positives [199]. Table 2 shows the advantages and disadvantages of the two bioreceptors. The advantages of aptamers as bioreceptors are the ability to target a variety of biomarkers, easily synthesized in large quantities and great stability, whereas high specificity and short time-consumption are the benefits of recognition based on immune response [198]. Different from the above bioreceptors, easily synthesized polymers containing specific molecular imprints for targeting SARS-CoV-2 are less susceptible to environmental factors and thus greatly improve sensor stability, yet such asynchronous sensors have not been extensively investigated [200]. The biosensors need to compensate for the disadvantages and develop further research directions to develop cost-effective POCT for the diagnosis of COVID-19. For another, nanotechnology is a common means to improve biosensor performance. For example, AuNPs are often used as labels in optical biosensors to improve biosensor sensitivity [176–179]. Nanomaterials in future research may function as substrates, receptors, signal transducers and powerful bio-binders thus becoming an integral part of biosensors. For portability, the combination of microfluidics and screen-printing technology make the biosensor detection platform smaller and more reliable even using a smartphone, making it more suitable for rapid detection in the field.

**Table 2:** The advantages and disadvantages analysis of Ag/Ab and aptamers as bioreceptors

Bioreceptor	Principle	Target	LOD	Advantages	Disadvantages	POC	References
Aptamers	Specificity of DNA aptamers	RNA and Ag	RNA: 44 ag/mL Ag: 37.5 pg/mL	Simplicity, flexibility, strong stability, and easily synthesized in large quantities	False positives, commercialized kits still need development	Clinics and laboratories	[133, 141, 142, 198, 199]
Ag or Ab	Immune response	Ab or Ag	Ab: 9.3 ag/mL Ag: 3.9 fg/mL	Simplicity, flexibility, speed, and high specificity	False positives, poor stability and commercialized kits still need development	Public, clinics and laboratories	[32, 43, 48, 50, 132, 153]

We have compared the above rapid detection methods for SARS-CoV-2 and the performance of the different biosensors (Table 3). For the detection of RNA, we found that NAAT-based methods were more sensitive with lower detection limits, but the biosensor-based method has the shortest time of 4 min, while for the detection of Ag and antibodies, the biosensor platform has both higher sensitivity and much shorter time (1 min). Therefore, the biosensor platform is far ahead of other methods in terms of time. In addition, many of the biosensor-based assays are capable of quantitative analysis of COVID-19, which is impossible with rapid diagnostic test kits.

**Table 3:** Comparing the performance of rapid detection methods for SARS-CoV-2

Types	Methods	Target	Details	LOD and ROD	Duration	References
Based on NAAT	RT-qPCR	RNA	DrectDetect (Absence of RNA extraction)	LOD: 1.67 copies/μL	1 ~ 2 h	[59]
	RT-qPCR	RNA	Integration with Big Data	–	3 ~ 4 h	[60]
	RT-qPCR	RNA	Water bath PCR and LFA	LOD: 8.44 copies/μL	45 min	[62]
	RT-qPCR	RNA	The SalivaDirect protocol and the Ubiquitome Liberty16 system	LOD: 12 copies/μL	1 h	[66]
	RT-qPCR	Variants RNA	Integration with ARMS	LOD: 1 copy/μL	2.5 h	[70]
	RT-qPCR	Variants RNA	Microfluidic chip-based	LOD: 10 copies/reaction	40 min	[73]
	RT-LAMP	RNA	Combined with CRISPR-Cas12	LOD: 1 copy/μL	32 min	[74]
	RT-LAMP	RNA	Particle imaging technique	LOD: 350 particles/ mL	35 min	[76]
	RT-HDA	RNA	Does not require thermal cycling	LOD: 6 copies/μL	2 h	[78]
	RPA	RNA	Integrated microdroplet array detection platform	LOD: 0.42 copy/μL	6–12 min	[80]
	PER	RNA	Combined with CRISPR-Cas12	LOD: 1.3 pM	40 min	[82]
Rapid diagnostic test kit	ELISA	Ab	Using microfluidic technology	–	–	[87]
	ELISA	Ab	Paper-based	LOD: 9 ng/µL ROD: 1 ng/µL-100 ng/µL	30 min	[88]
	ELISA	Ag	Double antibody sandwich method	LOD: 5 pg/µL	30 min	[89]
	LFIA	Ag	Depositing copper on the AuNPs-labeled LIFA test paper	LOD: 10 pg/mL	< 20 min	[91]
	LFIA	Ag(N)	Carboxy Gold Nanoshell-labeled	LOD: 156 pg/mL	< 15 min	[92]
	LFIA	Ag(N)	Colored cellulose nanobeads-labeled, double antibody sandwich method	LOD: 1 ng/mL	15 min	[93]
	LFIA	Ab	Selenium nanoparticles-labeled	LOD: 20 ng/mL	10 min	[94]
	LFIA	Ag(N)	AIE luminophores-labeled	LOD: 7.2 ng/mL	< 20 min	[95]
	LFIA	Ab	Ratiometric fluorescent analysis, Carboxyl-functionalized Europium chelate nanoparticles	LOD: 7.6 IU/mL ROD: 12.5-1000 IU/mL	15 min	[96]
	LFA	RNA	6-carboxyfluorescein-labeled, AuNPs capped with cysteamine as a control signal	LOD: 0.02 copy/μL	< 30 min	[97]
Electrochemical biosensors	Immunoimpedance biosensor	Ag(S)	Substrate: polyethylene terephthalate Modifiers: single-walled carbon nanotubes	LOD: 350 genome equivalents/mL	15 min	[128]
	Immunoimpedance biosensor	Ag(N)	Substrate: SPCE Modifiers: zinc oxide/reducedgraphene oxide	LOD: 21 fg/mL ROD: 1–10^4^ pg/mL	< 15 min	[53]
	Immunoimpedance biosensor	Ag(S)	Substrate: SPGE functionalized processed peptides as a reporter	LOD: 18.2 ng/mL ROD: 0.05–3 µg/mL	15 min	[131]
	Immunoimpedance biosensor	Ab	Modifiers: PPy-NTs/AuNPs	LOD: 0.386 ng/mL ROD: 0.4–8 ng/mL	< 1 h	[47]
	Immunoimpedance biosensor	Ag(S)	Substrate: SPCE Modifiers: SiO2@UiO-66	LOD: 100 fg/mL ROD: 100 fg/mL -10 ng/mL	< 5 min	[55]
	Electrochemical biosensor	Ab	Modifiers: colloidal quantum dots	LOD: 7.73 ng/mL ROD: 50–1250 ng/mL	< 1 min	[54]
	electrochemical biosensor	Ab	Substrate: GCE Modifiers: gold cluster	LOD: 9.3 ag/mL ROD: 0.1 fg-10 pg/mL	< 20 min	[50]
	Electrochemical biosensor	Ag(S)	Substrate: dual-gate oxide semiconductor thin-film transistor	LOD: 1.17 fg/mL ROD: 1 fg/mL -1 ng/mL	1 min	[132]
	Electrochemical biosensor	RNA	Substrate: SPCE Modifiers: gold nanoflowers combined with CRISPR-Cas13a	LOD: 4.4 × 10^–2^ fg/mL ROD: 10^–1^-10^5^ fg/mL	1.5 h	[133]
	ECL biosensor	RNA	Substrate: SPCE Ru(bpy)32 + -labeled	LOD: 0.1 fM ROD: 0.1 fM-10 µM	< 30 min	[135]
	Molecularly imprinted biosensor	Ag(RBD)	Substrate: SPCE Modifiers: molecularly imprinted polymer nanoparticles	LOD: 3.9 fg/mL ROD: 1 fg/mL-10 pg/mL	< 15 min	[48]
	BioFET	Ag(N)	based on an electrical double-layer gated BioFET system	LOD: 0.14 ng/mL ROD: 0.4–400 ng/mL	< 30 min	[140]
	BioFET	RNA	using flexible single-stranded DNA linked byrigid tetrahedral double-stranded DNA as a probe	LOD: 0.02 copy/μL	< 4 min	[142]
Optical biosensors	Colorimetric biosensor	RNA	AuNPs as a reporter, does not require sophisticated equipment	LOD: 0.5 ng	< 30 min	[175]
	Colorimetric biosensor	Ag(S)	Substrate: cotton swab orange nanopolymer-labeled	LOD: 100 pfu/mL ROD: 10^3^–10^8^ pfu/mL	5 min	[144]
	Colorimetric biosensor	M^pro^	bivalent peptide as a recognizer and AuNPs as a reporter	LOD: 18.9 nM	10 min	[51]
	Immunofluorescence biosensor	Ag(S)	hairpin structure of hACE2 mimetic peptide beacon as a reporter	LOD: 4.0 × 10^3^ pfu /test	< 3 h	[146]
	Optical biosensor	Ag(S)	Substrate: imprinted photonic crystal film low cost (approximately USD 1)	LOD: 429 fg/mL ROD: 1 pg/mL-100 ng/mL	< 15 min	[151]
	Optical biosensor	Ag(N)	Combined U-Bent plastic optical fiber with nanogold to immobilize the Ab	–	< 15 min	[152]
	SPR biosensor	Ag(S)	Using laser external differential feedback interferometry	LOD: 0.08 pg/ mL ROD: 10^–2^-10^3^ ng/mL	< 1 min	[153]
	LSPR biosensor	Ag(S)	Substrate: vertical microcavity Modifiers: nano-porous gold	LOD: 319 copies/mL	< 30 min	[52]
	LSPR biosensor	Ag(RBD)	Combined with optical imaging and artificial intelligence methods	LOD: 100 vp/mL ROD: 125.28-10^6^ vp/mL	< 12 min	[155]

Among the methods for rapid detection of SARS-CoV-2, the biosensor platform is a promising diagnostic method for future applications because it can accurately detect SARS-CoV-2 and variants in very short time and does not require expensive instrumentation and specialized technicians. Despite superior performance of the biosensor in all aspects, the problem of commercialization still exists. The cost of biosensor fabrication, the difficulty of mass production, and stability in use all need to be considered for widespread commercialization. Advances in nanotechnology within the last few decades have resulted in major improvements in electrochemical biosensing making them simple and efficient tools to measure the concentration of analytes and the detection of pathogens. Moreover, further work has been performed to miniaturize biosensors and make them portable, cost-effective, and reduce the sample size. These improvements have made electrochemical biosensors more and more attractive for developing POC tools with the help of development of nanotechnology [180]. More efforts have been put on improving sensitivity, enhancing portability, and reducing costs.

Compared with related categories [201–203], we focus on biosensors for rapid detection of SARS-CoV-2 based on nucleic acid amplification and LFIA to find novel directions from already established diagnostic technologies. The innovation of this review is the application, development and perspectives of nanomaterials in the rapid detection of biosensors. In particular, our perspective is more oriented towards the pragmatic application in the context of the COVID-19 pandemic, i.e., we compare and discuss already commercialized rapid test kits and describe emerging rapid tests with strong potential for commercialization. The application and development of diagnostic techniques in the context of a COVID-19 pandemic may provide a reliable reference for respiratory-transmitted viruses such as influenza virus, measles virus, and varicella-zoster virus. In fact, sensor platforms, which are considered to have great potential, have already been applied to the rapid detection of a variety of viruses other than SARS-CoV-2 [204, 205], and the novel sensing methods brought about by SARS-CoV-2 may provide new insights into the diagnosis of other diseases. We expect to accumulate experience in diagnostic techniques in the context of the COVID-19 pandemic in order to cope with future epidemics or outbreaks of novel viruses. Moreover, with the advancements in nanomaterial science and techniques, the novel coronavirus diagnosis will be updated in the coming time. The scope of development of a robust and rapid sensor for infectious diseases should be with successful commercialization and mass-scale production. In addition, the rapid assays of virus test results of patients could be recorded and researcher can monitor the health patients in real-time and take measures if necessary. It is conceivable that these biosensors will play a crucial role in controlling infectious diseases and public health. With this trend, the future will witness more breakthroughs and pioneering detection methods in attacking different viruses such as SARS-CoV-2.

## Data Availability

All data needed to evaluate the conclusions in this paper are either present in the paper or directly derived from the cited references.

## Abbreviations

RT-qPCR: Reverse transcription-quantitative polymerase chain reaction
Ct: Cycle threshold
NAATs: Nucleic acid amplification technologies
LFA: Lateral flow assay
Ag: Antigen
Ab: Antibody
S: Spike protein
E: Envelope protein
M: Membrane protein
N: Nucleoside protein
RT-LAMP: Reverse transcriptase loop-mediated isothermal amplification
RAT: Rapid antigen test
Cq: Cycle of quantification
VOCs: Volatile organic compounds
Mpro: Main protease
AuNPs: Gold nanoparticles
dPCR: Digital PCR
RT-HAD: Reverse transcription helicase-dependent amplification
PER: Primer exchange reaction
ELISA: Enzyme-linked immunosorbent assay
LFIA: Lateral flow immunoassay
RBD: Receptor binding domain
hACE2: Human angiotensin-converting enzyme 2
LOD: Limits of detection
SPCE: Screen-printed carbon electrode
GCE: Glassy carbon electrode
ECL: Electrochemiluminescence
BioFETs: Field-effect transistor-based biosensors
ST-NCF: Slightly tapered no-core fiber
SERS: Surface-enhanced Raman scattering
LSPR: Localized surface plasmon resonance
